# Case report: Conjunctival melanoma treated with relatlimab and nivolumab showing remarkable response

**DOI:** 10.3389/fonc.2024.1428152

**Published:** 2024-09-25

**Authors:** Mirona Attrash, Omar Badran, Yinon Shapira, Gil Bar-Sela

**Affiliations:** ^1^ Department of Oncology, Emek Medical Center, Afula, Israel; ^2^ Faculty of Medicine, Technion, Haifa, Israel; ^3^ Department of Ophthalmology, Carmel Medical Center, Haifa, Israel

**Keywords:** conjunctival melanoma, nivolumab/relatlimab, Opdualag, immunotherapy, cognitive health

## Abstract

Conjunctival melanoma, an uncommon form of ocular melanoma, shares some molecular characteristics with cutaneous melanoma and some with mucosal melanoma. Treatment of cases where it becomes advanced or metastatic raises unique treatment challenges. Nivolumab/relatlimab (Opdualag) recently received FDA approval for metastatic melanoma based on the phase 2/3 RELATIVITY-047 trial, which showed better median progression-free survival (PFS) in the first-line setting without new safety signals. The efficacy of this drug in conjunctival melanoma has not been reported yet.

**Case presentation:**

An 87-year-old woman with a history of mild dementia was admitted to the oncology department with a large, exophytic tumor protruding from her left eye, diagnosed as conjunctival melanoma two years previously. This tumor was secreting a whitish fluid and obstructing her vision. Immunotherapy with Opdualag was started, with a near clinical complete response after the 1^st^ cycle. The patient was treated with only four cycles due to worsening of her dementia.

**Conclusion:**

Nivolumab/relatlimab (Opdualag) is a promising treatment alternative in conjunctival melanoma when surgery is not viable.

## 1 Introduction

Conjunctival melanoma, an uncommon form of ocular melanoma, represents a challenging clinical scenario due to its aggressive nature and the potential for local invasion and metastasis (1). This malignancy, while sharing some molecular characteristics with cutaneous melanoma, poses unique treatment dilemmas, especially in cases where it becomes advanced or metastatic (2). The mainstay of treatment for early-stage disease is surgical excision, often complemented by adjuvant therapies. However, for advanced or metastatic conjunctival melanoma, systemic treatment options, including immunotherapy, have been explored with interest, reflecting practices in cutaneous melanoma management (3). in limited disease the treatment is still the surgery but due to tendency of recurring, radiation therapy is being an important part as adjuvant therapy (4).

The rationale for using immunotherapy, particularly checkpoint inhibitors targeting PD-1/PD-L1 and CTLA-4, stems from their success in treating cutaneous melanoma. These treatments have revolutionized the management of advanced melanoma, offering hope for similar efficacy in ocular melanomas, including conjunctival melanoma. Notably, pembrolizumab and the combination of nivolumab (a PD-1 inhibitor) and ipilimumab (a CTLA-4 inhibitor), known for its enhanced response rates in cutaneous melanoma, has sparked interest in its potential applicability for conjunctival melanoma (5).

Combining two immunotherapy treatments, such as anti-PDL-1 and anti-LAG3 (nivolumab with relatlimab), showed superior PFS over single-agent immunotherapy as a first line in unresectable melanoma patients (6). In our case, the patient had an impressive fungating mass protruding out of her orbit; to spare her orbital exenteration, a significant and disfiguring surgery, we decided to give the patient Opdualag (nivolumab with relatlimab) to induce a rapid response with minimal adverse effects.

Strength of the study: our study had several strengths like showing the positive effect of combined immunotherapy in this rare disease with remarkable results, on the other hand, with such shrinkage maybe Opdualag will be considered as an option for first line treatment in unresectable advanced conjunctival melanoma. Limitations of the study: there is limited data about this topic, and it is self-reported data.

## 2 Case presentation

An 87-year-old woman with a history of mild dementia presented to the ophthalmology department in August 2021 with noticeable changes in the anatomy and color of her left conjunctiva. A biopsy confirmed the diagnosis of invasive conjunctival melanoma. Given the patient’s age and the potential morbidity associated with surgical intervention, the decision of the patient and her family was not to pursue surgery.

**Figure 1:**
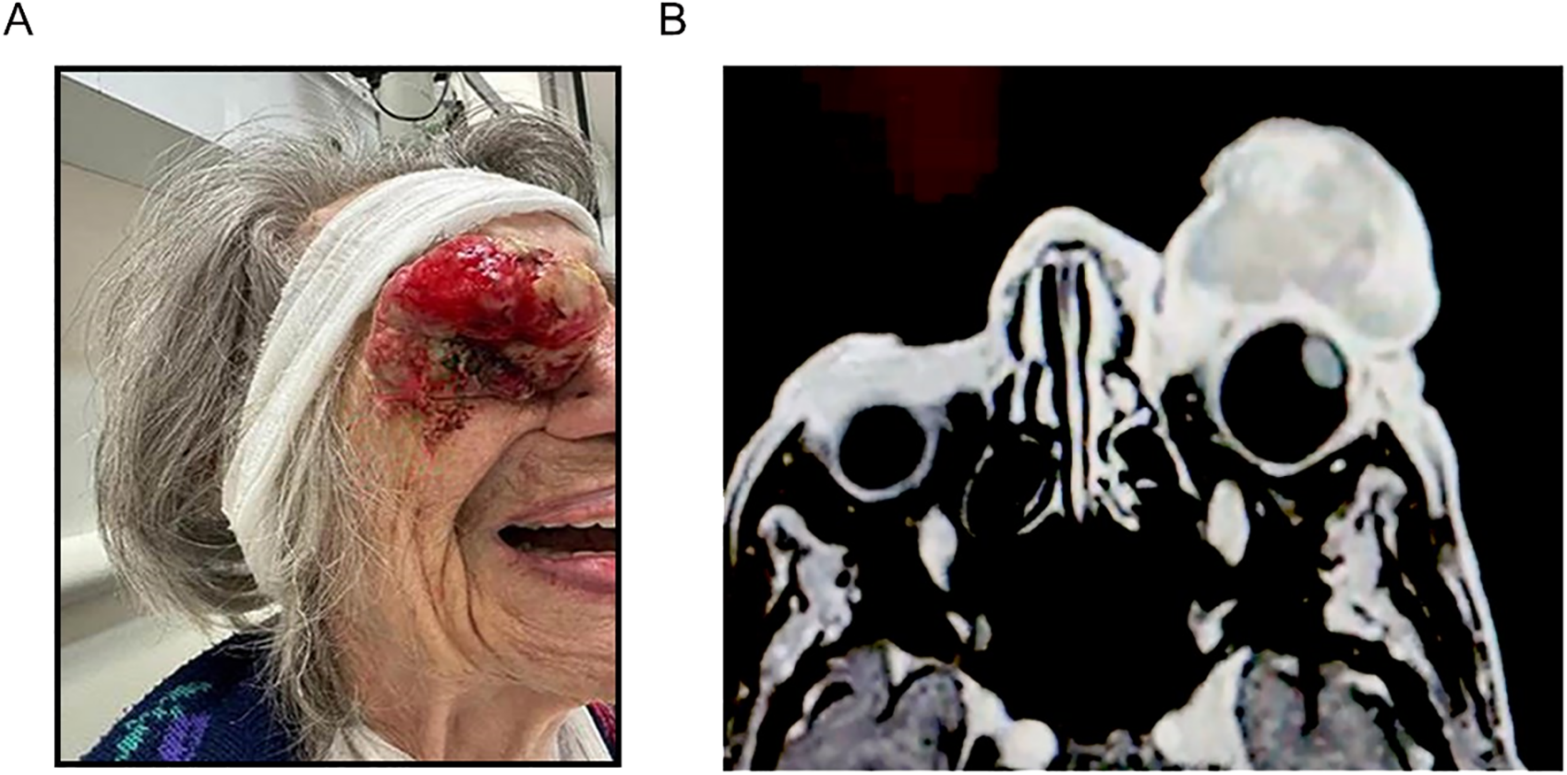
**(A)** The patient, before receiving treatment, showed a large, exophytic, fungating tumor protruding from her left orbit, known as a conjunctival melanoma. **(B)** Axial MRI T1 weighted with fat suppression showing the tumor.

Consequently, she did not receive any follow-up care until May 2023, when she was admitted with a large, exophytic, fungating tumor protruding from her left orbit. This tumor was obstructing her left vision and causing left eye pain and resultant insomnia (**Figure 1A**). MRI of the orbits showed a large, irregular, heterogeneously enhancing mass on the left eye’s anterior surface. The left globe was slightly irregular but overall preserved, with no evidence of posterior orbital extension (**Figure 1B**).

Comprehensive total body computer tomography (TBCT) performed at this time showed signs of chronic cerebral vascular incidents, a 2 cm meningioma near the left para-sellar area, slightly enlarged lymph nodes on both sides of the neck, perihilar lymph nodes, and two ground-glass opacity nodules in the right upper lung lobe. Crucially, there was no evidence of metastatic disease.

Given the extent of the tumor, the patient’s discomfort, and the effect on her quality of life, exenteration was presented as a palliative option. However, considering the patient’s age and cognitive condition, and after counseling with the patient’s family, a multidisciplinary team decided against surgical intervention. Instead, it was decided to initiate treatment with a combination of nivolumab/relatlimab, known for its higher response rate than single-agent immunotherapy and lower side effects than other immunotherapy combinations.

**Figure 2:**
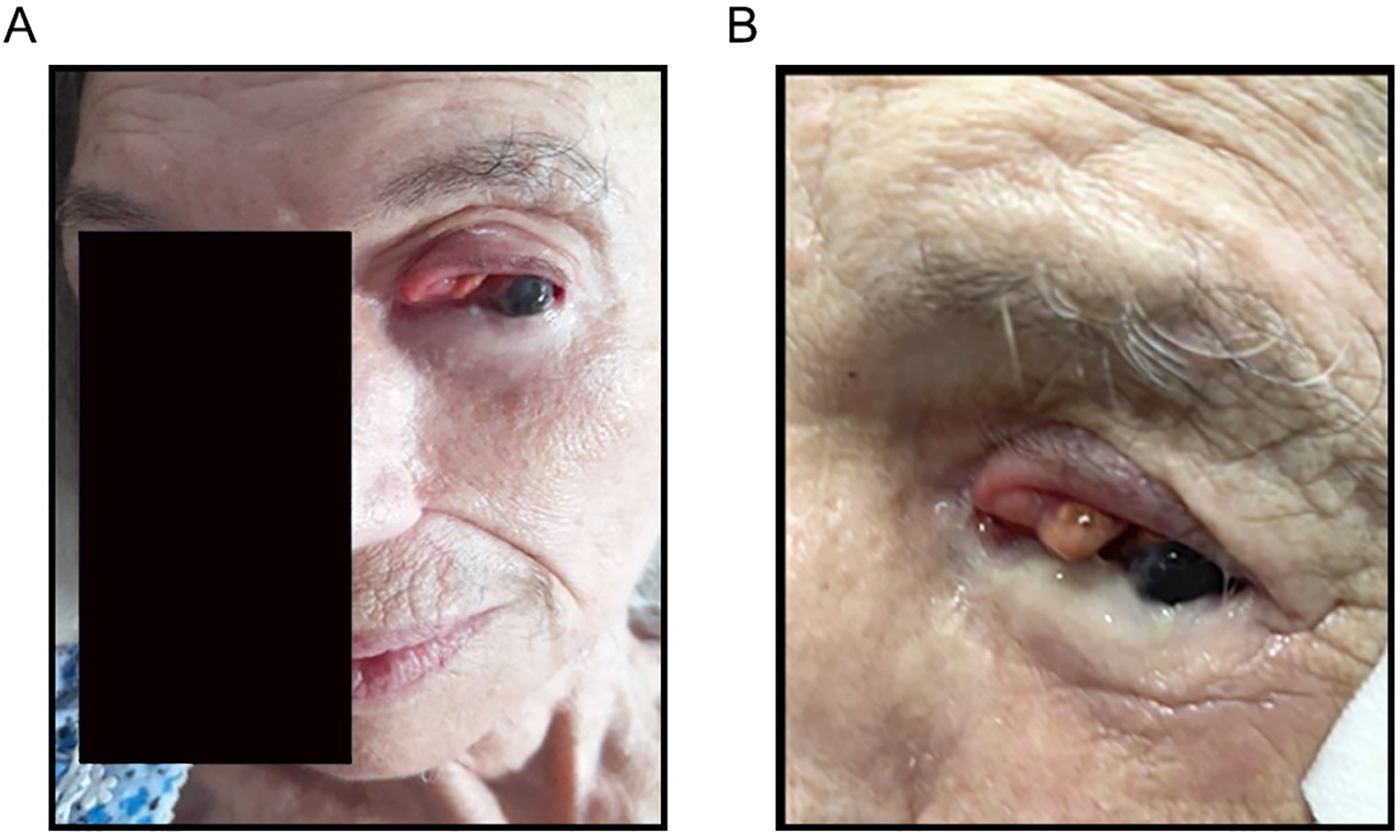
**(A)** Withdrawal of most of the tumor after receiving one course of Opdualag. **(B)** Near complete remission of conjunctival melanoma after receiving the third treatment of Opdualag.

Treatment commenced in June 2023 at HaEmek Medical Center, Israel. Remarkably, the tumor mass disappeared just one month after the first dose, demonstrating a dramatic clinical response (**Figure 2A**).

However, the patient’s cognitive state worsened significantly after the third treatment dose. Corticosteroid therapy with Prednisone was initiated to manage her symptoms. After tapering off the corticosteroids and administering the fourth dose of nivolumab/relatlimab, further treatment was halted due to further worsening of her cognitive state. Upon completing the treatment cycles, a reevaluation revealed clinically a tiny residual tumor. However, in line with the patient’s wishes and considering her general condition, it was decided that it was preferable to avoid surgery. (**Figure 2B**). The patient continued living in her home without further treatment.

## 3 Discussion

In the context of our elderly patient, who also suffered from dementia, the decision to initiate immunotherapy underscores the need for careful consideration of both the potential benefits and the risks, particularly given the limited literature on immunotherapy outcomes in patients with pre-existing cognitive impairment. The case highlights the necessity of individualized treatment planning and the importance of ongoing research to understand better the implications of immunotherapy in this unique patient population. Nonetheless, after careful consideration with the patient’s family, this treatment spared the patient a large, complex, and disfiguring surgery, the only other palliative alternative to trial with immunotherapy.

The rationale behind combination therapy revolves around the synergistic potential of dual checkpoint inhibition to enhance the anti-tumor immune response. The Opdualag combination, targeting both the PD-1 and LAG-3 pathways, has been investigated for its efficacy in melanoma, showing promising results. Studies in cutaneous melanoma have demonstrated that such combinations can lead to higher response rates compared to single-agent immunotherapy, albeit with an increased risk of immune-related adverse events (irAEs).

The literature on the efficacy of immunotherapy, particularly combination therapy, is sparse but growing in the case of conjunctival melanoma. The decision to employ Opdualag was influenced by its effectiveness in cutaneous melanoma, hypothesizing that its benefits could extend to conjunctival melanoma, a malignancy with limited treatment options once it progresses beyond local therapies.

In the RELATIVITY 047 trial, 714 patients received immunotherapy for unresectable cutaneous melanoma; 359 received nivolumab alone as a single agent, and 355 received combined therapy with Opdualag. The median progression-free survival with Opdualag was 10.1 months, whereas with nivolumab alone, it was 4.6 months, with a 10.3 percentage point difference in the objective response rate. Grade 3-4 adverse events occurred in 18.9% of the Opdualag group and 9.7% of the nivolumab group. The response rate and progression-free survival were significantly higher in the Opdualag group than in single-agent nivolumab. Our patient received Opdualag based on this data.

However, immunotherapy treatments can lead to neurological side effects, especially peripheral complications, which, although rare, can be fatal. The study by Sara Mancone et al. showed that 5 of 55 patients who received immunotherapy treatment developed neurological irAEs grade 3-4. There was no relevant data regarding irAEs and dementia. It is not well known if dementia is a direct side effect of immunotherapy or if there is a regression in the patient’s daily activities due to weakness secondary to irAE (7). Therefore, combination therapy in patients with dementia presents additional challenges. In the case of our patient, the progression of dementia following the initiation of immunotherapy raises important questions regarding the safety and tolerability of these agents in individuals with pre-existing cognitive impairment.

The worsening of dementia in this context was probably related to the patient’s general health and was not a direct immune-related side effect. The literature on immunotherapy in patients with pre-existing neurological disorders is limited, highlighting a critical area for further research. Understanding the interactions between immunotherapy and neurological conditions is essential to optimize treatment strategies and manage potential complications effectively.

Regarding alternative treatments, radiation therapy, including brachytherapy, photon-beam, and proton therapy, is often considered an alternative or adjunct to surgical treatment in conjunctival melanoma. These treatments can be particularly beneficial in preserving the eye and maintaining better cosmetic and functional outcomes than extensive surgery (8).

Additionally, combining radiotherapy with immunotherapy is a promising idea. Hypofractionated radiotherapy, such as SABR, has shown improvements in local control and induced an abscopal effect, where localized treatment also reduces distant metastases. A study by Lo Greco demonstrated that combining high-dose radiotherapy with the immune checkpoint inhibitor cemiplimab improved efficacy in advanced cutaneous squamous cell carcinoma (4). However, this combination has not been tested in conjunctival melanoma and thus was not considered for integration in this context.

## 4 Conclusions

To our knowledge, the current case is the first reported patient with conjunctival melanoma treated with nivolumab/relatlimab (Opdualag). The case showed this immunotherapy combination’s high efficacy in treating a complex conjunctival melanoma case, sparing primary surgical intervention critical in elderly patients with additional health considerations. The possible correlation between immunotherapy and the worsening of dementia should be studied in real-world data reports.

## Data Availability

The original contributions presented in the study are included in the article/supplementary material. Further inquiries can be directed to the corresponding author.
